# A new disjunct species of *Eriolaena* (Malvaceae, Dombeyoideae) from Continental Africa

**DOI:** 10.3897/phytokeys.111.29303

**Published:** 2018-11-06

**Authors:** Laurence J. Dorr, Kenneth J. Wurdack

**Affiliations:** 1 Department of Botany, MRC-166, National Museum of Natural History, Smithsonian Institution, P.O. Box 37012, Washington, D.C. 20013-7012, USA National Museum of Natural History, Smithsonian Institution Washington United States of America

**Keywords:** Dombeyoideae, *
Eriolaena
*, Malvaceae, Mozambique

## Abstract

*Eriolaenarulkensii* Dorr, **sp. nov**. is described and illustrated. This attractive shrub is endemic to coastal Mozambique. The new species has apically winged seeds, which place it in a group of Malvaceae (Dombeyoideae) that is found in Asia and Madagascar and which had not previously been found in continental Africa.

## Introduction

When A.J.H. (Ton) Rulkens encountered a yellow-flowered shrub on the shore of Pemba Bay (Baia de Pemba) near the city of Pemba in north-eastern Mozambique, he could not identify it and sent photographs to John E. Burrows (BRNH) who also was unsure as to its identity. Burrows, in turn, shared photographs of the plant with several botanists including I. Darbyshire (K) who identified it as a species of Dombeyoideae (Malvaceae). Darbyshire noted that it did not match either *Dombeya* Cav. or *Melhania* Forssk., the two genera of Dombeyoideae known from the area (Wild and Gonçalves 1979; Cheek and Dorr 2007), and he observed that it closely resembled *Helmiopsis* H. Perrier, a genus of Dombeyoideae endemic to Madagascar. The lack of scales on the unidentified plant, however, led Darbyshire (personal communication) to doubt whether or not it belonged in that genus. Independently, photographs were sent to one of us (LJD) with a simple request for confirmation that the species belonged in the “Sterculiaceae” (i.e. Malvaceae sensu lato). An initial scepticism about this placement very quickly gave way to the realization that the photographs depicted a species that belonged to a genus of Malvaceae (Dombeyoideae) that had not yet been reported from continental Africa. More specifically, the new species appeared to belong to one of two endemic Malagasy genera, either *Helmiopsis* or *Helmiopsiella* Arènes.

One of us (LJD) identified the new species provisionally as a new species of *Helmiopsiella*, a genus of four species endemic to Madagascar (Barnett 1988a). This determination was based solely on photographs that bore a remarkable but, in retrospect, superficial resemblance to *Helmiopsiellapoissonii* (Arènes) Capuron ex L.C. Barnett. Morphological characters that distinguish *Helmiopsiella* and *Helmiopsis* are weak (see also Skema 2012) and the attempt to place the new species from Mozambique underscored this problem. In any case, the provisional determination was adopted and it is used in Burrows et al. (2018; “*Helmiopsiella* sp. A”), on a Flickr website maintained by Rulkens (https://www.flickr.com/photos/47108884@N07/; “*Helmiopsiella* sp. Pemba”) and in an on-line version of the flora of Mozambique (Hyde et al. 2018; “*Helmiopsiella* sp. A”). However, once herbarium material was received and a molecular phylogenetic analysis was initiated (Dorr et al. in prep.), it became apparent that a better generic placement was with the Asian genus *Eriolaena* DC. The need to have a name for this endangered shrub, which can be used for checklists and conservation reports, leads us to validate *E.rulkensii* in anticipation of publishing a more comprehensive molecular phylogenetic study of the wing-seeded taxa of Dombeyoideae.

## Taxonomic treatment

### Eriolaena rulkensii

Taxon classificationPlantaeMalvalesMalvaceae

Dorr sp. nov.

urn:lsid:ipni.org:names:60477347-2

Figures 1 , 2

Helmiopsiella sp. A: Burrows et al., Trees shrubs Mozambique: 590. 2018.

#### Diagnosis.

Differs from *Eriolaenawallichii* DC. in having entire or sparingly toothed epicalyx bracts (versus laciniate epicalyx bracts) and an androecium of 10–15 anthers alternating with 5 staminodes (versus an androecium comprised of numerous stamens and no staminodes).

#### Type.

Mozambique. Cabo Delgado: Pemba, close to Pemba Bay, near Chibuaburare, 12°58'26"S, 040°30'10"E, 9 m alt., 23 Feb 2014 (fl, fr), *A.J.H. Rulkens 1* (holotype: US-01184177; isotypes: BNRH, K-0001291030, K-0001291031, LMA, US-01184178).

#### Description.

*Shrubs* or straggly trees, 2–6 m tall. Bark smooth, mottled grey and brown; young stems with scattered, appressed multi-radiate stellate hairs; older stems lenticellate, ± glabrescent. *Leaves* simple, alternate, petiolate, stipulate; leaf blades ovate to broadly ovate, 7–12.5(–14) cm long, 5–9(–9.5) cm wide, apices long acuminate, bases cordate to truncate, margin coarsely crenate except base of blade entire, (3–)5-nerved from the base, primary, secondary and tertiary nerves clearly visible below, veinlets visible below with 10× magnification, ± glabrous above and below except for scattered minute multi-radiate stellate hairs that are more numerous on the primary and secondary nerves and toward leaf base, somewhat lustrous above, matt below; domatia absent; petioles 3–4.5(–7) cm long, sparingly pubescent with scattered minute multi-radiate stellate hairs; stipules long acicular, 9–12(–15) mm long, ca. 1 mm wide at base and tapering to 0.25 mm below apex, sparingly pubescent with scattered minute multi-radiate stellate hairs, caducous. *Inflorescence* paniculate, axillary and terminal, lax, 20–27 cm long, 20–25 cm wide; pedicels to 9(–10) cm long. *Epicalyx* bracts 3, acicular, 7–10 × 2 mm, entire or sparingly toothed apically, ± evenly spaced around the axis in bud, but clustered on one side at anthesis, caducous. *Calyx* 5-lobed, valvate, shortly (1.5–2 mm) connate at base, lobes lanceolate, 7–8 × 2 mm, apices acute, somewhat thickened distally, sparingly pubescent externally with appressed, minute multi-radiate stellate hairs, glabrous internally, smooth (i.e. nerves not visible). *Petals* 5, broadly obovate, 14–16(–22) mm long, 14–15(–20) mm wide, ± symmetrical, apices crispate, bases cuneate, bright yellow in vivo, glabrous externally and internally. *Androecium* of 10–15 anthers alternating with 5 staminodes; anthers in an outer whorl, borne in fascicles of 2(3), common filaments ca. 3–3.25 mm long, glabrous; anther sacs 2–2.25 mm long; staminodes in an inner whorl, ligulate, 10 × 1 mm, glabrous. *Style* 1, ca. 4 mm tall; stigmas 10, recurved apically, pale yellow to white. *Fruit* a loculicidally dehiscent capsule, obovoid, ± 1.5 cm in diameter, 10-ridged, sparingly pubescent with scattered minute multi-radiate stellate hairs, eventually splitting into separate mericarp-like structures. *Seeds* 1(2) per locule, obovate, 4 × 2 mm, laterally flattened, glabrous, each seed with a narrow, ca. 1 mm wide, hyaline, dorsal and apical wing.

**Figure 1.**
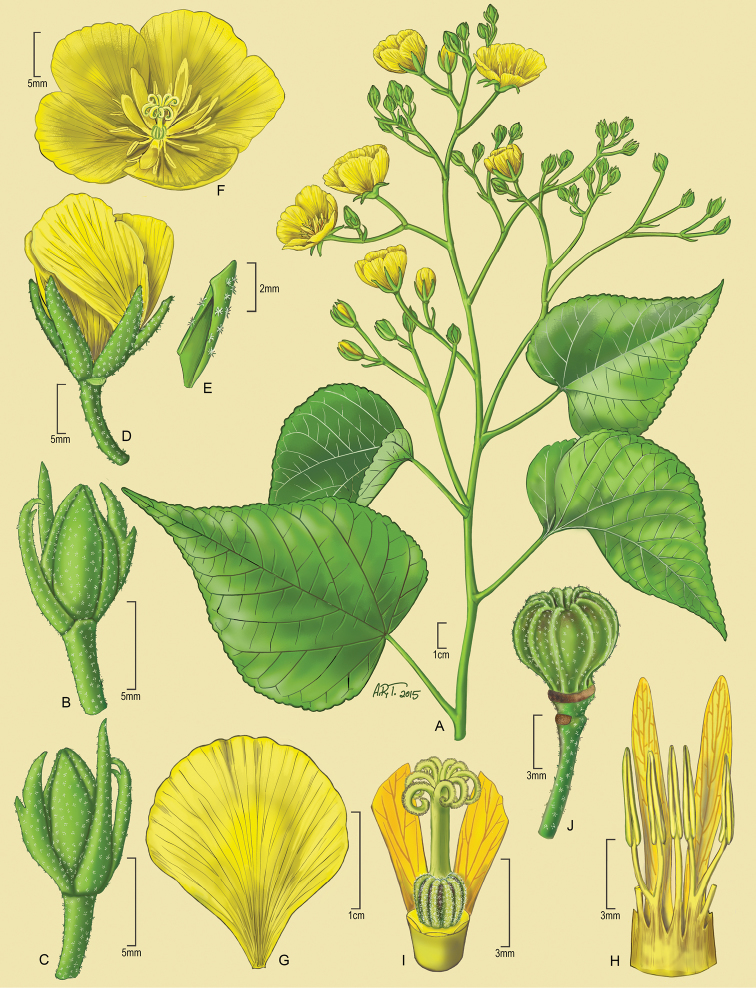
*Eriolaenarulkensii*. **A** Habit **B, C** Flower buds with 3 epicalyx bracts each **D** Immature flower with petals (yellow), sepals (green) and scar of one dehisced epicalyx bract **E** Detail of sepal **F** Flower at anthesis showing petals, anthers, staminodes and gynoecium **G** Detail of petal **H** Detail of androecium showing androecial tube, anthers in fascicles and staminodes **I** Gynoecium and base of two staminodes **J** Immature capsule showing scars from dehisced calyx lobes and epicalyx bracts. (Source: *A.J.H. Rulkens 1*, US).

**Figure 2.**
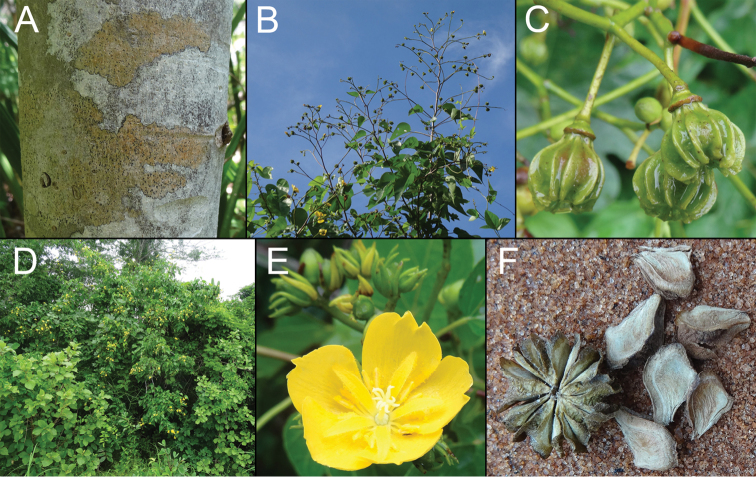
*Eriolaenarulkensii*. **A** Trunk and bark **B** Infructescence **C** Immature capsules with prominent ridges **D** Habit (shrub in centre with yellow flowers) **E** Flower (foreground) with stamens in fascicles of 2(3), each alternating with a ligulate staminode, and a simple style with 10 stigmas; flower buds (background) with 3 epicalyx lobes subtending each calyx **F** Capsules (mature) with loculicidally dehiscent mericarp-like structures. Photographs: A.J.H. Rulkens.

#### Etymology.

Named for A.J.H. (Ton) Rulkens, an agronomist who works for OXFAM Belgium to strengthen small-scale farmer organizations in Cabo Delgado province, Mozambique. Rulkens is also a keen amateur botanist and photographer who has made many interesting plant discoveries in Mozambique, especially amongst representatives of the succulent flora (McCoy et al. 2014, 2017; McCoy and Baptista 2016).

#### Distribution and ecology.

Endemic to northern Mozambique where it is known from several localities on the eastern and southern shore of Pemba Bay (Baia de Pemba) near the city of Pemba where it occurs on heavy clay over coral-rag in coastal scrub at the upper margin of mangrove communities (Burrows et al. 2018); ca. 10 m alt. According to Rulkens (personal communication), Ernst Schmidt observed the shrub between Macomia and the coast in 2009 but later discovered the plant had been cut down. Rulkens also observed additional locations with many plants about 10 km from Pemba in small patches of coastal forest on fossil coral substrate.

#### Threats.

*Eriolaenarulkensii* is exploited for firewood (fide *Rulkens 1*, in sched.) and, on a different scale, it is threatened because the coastal forests and woodlands of northeast Mozambique are subject to increased development following the instability resulting from the independence and civil wars (Timberlake et al. 2011). *Eriolaenarulkensii* is only one of many new species discoveries and new records from the Cabo Delgado area of Mozambique, others of which are enumerated by Timberlake et al. (2011).

## Discussion

Within the Dombeyoideae, Barnett (1988b) recognized morphological and anatomical similarities that united the Asian *Eriolaena* and Malagasy *Helmiopsis* and *Helmiopsiella* as a group. All three genera have woody and ovoid- or obovoid-conical capsules as well as seeds with apical wings. Skema (2012) subsequently published molecular data that support the distinctive nature of this “winged-seed clade” and demonstrated that the clade was early diverging from *Dombeya* sensu stricto. *Eriolaenarulkensii* represents the first species of this “winged-seed clade” to be found in continental Africa.

*Eriolaenarulkensii* is not the only example of an Asian/Malagasy species that also occurs in Mozambique but is otherwise absent from continental Africa. *Dianellaensifolia* (L.) DC. (Asphodelaceae) is widespread around the Indian Ocean, but confined in continental Africa to the foothills of a few mountain ranges in Mozambique (Hyde et al. 2018). Additionally, there are numerous examples of genera, both plants and animals, found in Asia, Madagascar and continental Africa (Schatz 1996; Renner 2004; Warren et al. 2010).

## Supplementary Material

XML Treatment for Eriolaena rulkensii
